# 

**Published:** 1884-03

**Authors:** 


					﻿1884
OLD SERIES
{ Vol. XXV.
1855 o
NEW SERIES
Vol. I—No. 1.
WTMffif

(JOURNAL

3$WbJ
W.F.WESTMORELAND, N®.■
H.V.M.MILLER,M.D.LL .D.W
JAMES A.GRAY. M.D."
Published By james p.harrison&.cq^
Atlanta ,ga. 4a
SUBSCRIPTION: 2.50 A YEAR.
				

